# A Keynote Address: A Guide to Supporting Conference Speakers with Lived Experience of Cleft Lip and/or Palate

**DOI:** 10.1177/10556656231211684

**Published:** 2023-11-06

**Authors:** Kenny Ardouin, Danielle McWilliams, Amanda Bates

**Affiliations:** 1 School of Psychology, Speech & Hearing, 2496 University of Canterbury, Christchurch, New Zealand; 2 Salomon's Institute for Applied Psychology, 2238 Canterbury Christ Church University, Canterbury, UK; 3 Centre for Health Services Studies, University of Kent, Canterbury, UK

**Keywords:** psychosocial adjustment, quality of life, cleft lip and palate

## Abstract

Although the value of diversity within academia and society is increasingly recognised, the role of speakers with lived experience at cleft and craniofacial conferences remains inconsistent. This perspectives article shares reflection from three academics with lived experience of cleft discussing the value of including lived experience speakers routinely within conferences and outlining common challenges and barriers to the involvement of “experts-by-experience”. Key considerations and recommendations are offered to help conference organisers and delegates to make the most of the lived experience perspective, while ensuring the conference experience is positive for lived experience speakers.

## Background

In recent years, the necessity to represent those communities affected by policy decisions has become increasingly apparent, both within academia and society. One area in which representation has markedly evolved is healthcare. While the experience, expertise, and education of clinicians and other health professionals remains paramount to positive patient outcomes, even the most academically qualified person in the room is no longer required to make clinical decisions alone. Instead, people with lived experience (sometimes known as “experts-by-experience”) are considered active collaborators in healthcare. A person's experience of living with their condition crafts constellations of knowledge and understanding that are unique and crucial in decision-making, research, and evaluation. Only through lived experience can a person accurately describe the sensations experienced within their body: no diagnostic imaging or clinical assessment tool can predict or measure such experiences. Lived experience expertise has the benefit of being able to make connections between treatment and outcomes that experts by training may not even know to explore. Only those living with a particular condition can accurately describe the psychological experiences of an individual as the same situations that develop resilience in some, create feelings of overwhelm or despair in others. Inclusion of patient perspective in patient-centred care is established for individual-level care, and people with lived experience increasingly contribute to policy decisions. For example, in the United Kingdom, conferences about homelessness routinely feature experts-by-experience – who are provided training beforehand to enable their full participation,^1^ and their voices are represented and incorporated into National Health Service (NHS) policies related to healthcare provision for people who are homeless.^1^

An increasing number of health professionals, researchers, and academics with lived experience of cleft lip and/or palate are entering the workforce where our insights have the potential to encourage crucial discussions. Despite this, the perspective of those with lived experience remains underrepresented at cleft and craniofacial conferences.

## Consequences of the Endemic Lack of Lived Experience at Cleft and Craniofacial Conferences

Recently, diversity within academia is increasingly emphasised and recognised as necessary.^2,3^ Progress is underway to identify diversity gaps and facilitate inclusion of under-represented groups at conferences, including women,^4,5^ people of colour,^6^ and the LGBTQIA+ community.^7^ Despite promotion of diversity, inequities persist for people with cleft and craniofacial conditions, as for other disabilities.^8^

Even within cleft workplaces, a lack of representation persists. At a recent global cleft conference, significant expenditure was allocated to ensure robust representation of low-and-middle-income countries (LMIC), which greatly increased the diversity of pedagogies at the conference. Similar supports were not extended to people with lived experience; rather, their attendance was mostly coincidental (ie, a researcher presented at the conference, and also had lived experience). These authors were invited to deliver a panel discussion around lived experience of cleft, and appreciated the opportunity to share our perspective at such an important forum. The panel was well-received, with reports that it was a ‘highlight’ and a topic attendees would appreciate hearing discussed more often. The suggestion that lived experience would make a valuable plenary session is one we endorse. The myriad of positive feedback – and unconcealed surprise at the value of our perspective – highlights how rarely such a platform avoids resembling tokenism.

We are pleased the discussion resonated with attendees, as we felt future inclusion of lived experience relied on our panel's success. Our desire to promote candid sharing of lived experiences conflicted with the pressure to avoid jeopardising future opportunities for experts-by-experience. We anticipate a day when lived experience is so routinely presented at conferences that such topics are considered standard inclusion to enrich, challenge, and sit equal to contributions by clinicians and researchers.

Representing lived experience at events can be both exciting and – at times – disheartening, where logistical and emotional support are insufficient. For example, these authors have each previously self-funded and organised their own travel and lodging: limited personal budgets led to inadequate housing during conference attendance.

These issues are commonplace across conferences. At another 2022 conference, one author delivered the keynote address, and was later shocked when the Chairperson of the national cleft support group indicated themselves as “the only person in the room representing patient voice” – a comment not only dismissing the author's expertise-by-experience, but also equating *parent* voice with *patient* voice, a hurtful and invalidating conflation of experiences. This experience reinforces that one is seen as either a person with lived experience, *or* a researcher – never both – a phenomenon also recognised in other areas of healthcare.^9^ In reality, many of us wear multiple hats, and we all hold unique perspectives from straddling a variety of spheres, including both professional and personal. These multidimensional perspectives are all valid.

## The Value of Lived Experience at Conferences

People with lived experience offer valuable insight acquired through managing a condition across a lifetime's worth of settings. By including lived experience expertise at cleft or craniofacial conferences, delegates receive a more genuine, holistic picture of cleft. Involving experts-by-experience early during conference planning is also advisable, to ensure lived experience remains prominent within the programme, and that the conference is welcoming for people with lived experience. For example, experts-by-experience can draw attention to choices like the frequency of medical topics versus psychology topics, and inform decisions related to conference policies on inclusive language, provision of suitable catering for participants with cleft, appropriately accessible venues, and conversation topics that will not inadvertently cause psychological distress. Experts-by-experience continued involvement during and after the conference further normalises the presence of lived experience at conferences, and ensures these perspectives are reflected in post-conference discourse. Executed well, working with experts-by-experience enhances the quality, relevance, and accessibility of conferences.

## Key Considerations

Exclusively seeking input from people with lived experience who are already known to academic and healthcare communities could potentially bias lived experience representatives towards being highly educated, articulate, and outwardly well-adjusted. While often an appropriate choice, it is prudent to recognise these individuals cannot speak on behalf of everyone with cleft.

To encourage diversity of experts-by-experience, it is worth investing in conference accessibility measures. Dedicated funding to cover the costs of registration, transport, and lodging for people with lived experience would be a strong candidate for consideration. People with lived experience provide valuable insight throughout the conference lifecycle but often fund their own travel and lodging (as was the case for these authors on various occasions). Ideally, experts-by-experience would be valued as much as ‘experts-by-training’ and would receive equitable financial assistance for attending. Unlike most delegates, whose transport and lodging costs are typically covered by employers, people with lived experience may not have employer funding available, so a bursary would be facilitative. Additionally, people with lived experience may need to take leave to attend, increasing their financial burden. Left unaddressed, these barriers limit the ability of people with lived experience to attend conferences. In addition, the disparity of resources can serve to further alienate individuals with lived experience, and reduce representation.

Additional emotional support would be another valuable consideration for presenters with lived experience. Though sharing personal experiences at a conference can feel empowering, it requires vulnerability which can be exhausting and/or distressing. Attending a conference as an expert-by-experience may entail attending without peers, which can feel isolating and could compound existing social anxieties resulting from visible and/or speech differences. Organisers should be mindful of this and ask invitees in advance what support they might need, including catering or accessibility requirements (eg, the need to be seated when presenting and access to a quiet space). The opportunity to access support services during the conference period could be beneficial – including identifying support groups that are also attending, opportunities to socialise with other experts-by-experience, or access to more formal support such as psychological services. Preparing for accommodations prevents embarrassing or awkward situations. Further, it is appreciated when conference organisers check in with speakers before their departure, and recommended that invitees are contacted in the week following the event for debrief, to check for continued positivity about the event, and the opportunity to provide feedback.

Finally, people with lived experience who work in cleft may self-censor to manage conflicting personal insights and professional relationships. Despite striving for authenticity and honesty, sharing personal experiences at a conference of your professional peers carries pressure to curate a distinctly flattering narrative. Though we are at a loss to suggest a tangible remedy for such internal conflicts, perhaps keeping the following in mind is enough: whilst experts-by-experience wear multiple hats, they must all sit on the same head.

## Facilitating Positive Interactions

The best way to interact with people with lived experience is simple – just interact with us! Like you, we find being alone in a crowded room uncomfortable – so please say hello, introduce yourself, and ask about what we do. Like most conference attendees, we like discussing our work and our presentation topics. Preferably refrain from making personal comments, especially related to appearance. Regardless of intention, comments such as “your repair is incredible – who did your surgery?” can catch us off-guard. If you want to compliment us, please compliment *our* work – not someone else's repair job! Having people with lived experience present early in the conference can foster an inclusive tone for the event, and organisers may consider sharing guidelines for appropriate language use, and respecting attendees’ privacy (eg, not using a conference as the time to discuss treatment options with a delegate who also happens to be your patient).

## Summary

We have collated some thoughts for organisers to make the most of the lived experience perspective and make conferences positive events for speakers.

### Equitability Considerations

- Have people with lived experience been included in conference planning?
- Are accessibility needs (eg, suitable catering, option to present while seated, etc) noted and addressed?
- Are financial barriers/burdens addressed?
- Is this person paid consistently with other speakers?
- Does travel and lodging need arranging?
- Is the environment welcoming for someone with lived experience, or solely oriented towards another group?
- Does this person have upcoming treatment, or are they recovering from previous treatment which may influence their ability to participate fully?
- How does the invited speaker feel about participating in Q&A?
- Have we spoken directly with this person to ensure their concerns and needs have all been addressed?

### Emotional Support Considerations

- How can I alleviate any concerns the person may have prior to attending?
- Does the person know what to expect at the conference, and what is expected of them?
- What support options are at the conference for people with lived experience?
- Who will check in with the speaker during the conference?
- How will I debrief with the speaker after the conference?

## Future Directions

We hope to have provided some insights and discussion points for making conferences more equitable for experts with lived experience. More research into the intersection of working and living with cleft and craniofacial diagnoses is imperative. Additionally, better evidence for how best to supervise people with lived experience when they work academically or clinically in the field of their condition is welcomed. Such research would acknowledge the unique perspectives and challenges associated with simultaneously living and working with cleft and craniofacial conditions.
